# Function and diversity of P0 proteins among cotton leafroll dwarf virus isolates

**DOI:** 10.1186/s12985-015-0356-7

**Published:** 2015-08-12

**Authors:** Renan S. Cascardo, Ighor L. G. Arantes, Tatiane F. Silva, Gilberto Sachetto-Martins, Maité F. S. Vaslin, Régis L. Corrêa

**Affiliations:** Department of Genetics, Federal University of Rio de Janeiro, Rio de Janeiro, Rio de Janeiro Brazil; Department of Virology, Federal University of Rio de Janeiro, Rio de Janeiro, Rio de Janeiro Brazil; Present address: Departamento de Biotecnologia, Escola de Engenharia de Lorena, Universidade de São Paulo, Lorena, São Paulo Brazil

## Abstract

**Background:**

The RNA silencing pathway is an important anti-viral defense mechanism in plants. As a counter defense, some members of the viral family *Luteoviridae* are able to evade host immunity by encoding the P0 RNA silencing suppressor protein. Here we explored the functional diversity of P0 proteins among eight cotton leafroll dwarf virus (CLRDV) isolates, a virus associated with a worldwide cotton disease known as cotton blue disease (CBD).

**Methods:**

CLRDV-infected cotton plants of different varieties were collected from five growing fields in Brazil and their P0 sequences compared to three previously obtained isolates. P0’s silencing suppression activities were scored based on transient expression experiments in *Nicotiana benthamiana* leaves.

**Results:**

High sequence diversity was observed among CLRDV P0 proteins, indicating that some isolates found in cotton varieties formerly resistant to CLRDV should be regarded as new genotypes within the species. All tested proteins were able to suppress local and systemic silencing, but with significantly variable degrees. All P0 proteins were able to mediate the decay of ARGONAUTE proteins, a key component of the RNA silencing machinery.

**Conclusions:**

The sequence diversity observed in CLRDV P0s is also reflected in their silencing suppression capabilities. However, the strength of local and systemic silencing suppression was not correlated for some proteins.

**Electronic supplementary material:**

The online version of this article (doi:10.1186/s12985-015-0356-7) contains supplementary material, which is available to authorized users.

## Background

Cotton leafroll dwarf virus (CLRDV) is the causal agent of an economically important cotton (*Gossypium hirsutum*) disease called cotton blue disease (CBD) [1]. *Aphis gossypii*-transmitted CBD has been observed in several cotton-producing areas of Central Africa, Asia and South America [2]. CBD symptoms are characterized by stunting, leaf rolling, vein yellowing, dark-green leaves and small bolls, leading therefore to severe yield losses when aphid populations are not properly controlled. In Brazil, CBD is present in almost all cotton growing fields and the disease was also partially controlled by the application of insecticides to decrease aphid populations and by the use of CBD-resistant cotton cultivars. Since 2006, several resistance breaking CLRDV isolates have been observed throughout the country, producing CBD-like symptoms in formerly resistance cotton lines [3]. Apart from typical CBD symptoms, resistant or susceptible cotton varieties infected with CLRDV resistance-breaking isolates may also display reddish and withered leaves. Resistance breaking isolates are now widely distributed in Brazilian cotton growing areas, making the use of insecticide for aphid control compulsory.

The CLRDV genome resembles a typical member of the genus *Polerovirus*, family *Luteoviridae* and contains six open reading frames (ORF0 to ORF5) [4]. The genome is divided into two gene-containing portions, separated by an approximately 200 nucleotides intergenic region. Three open reading frames (ORF3, ORF4 and ORF5) are located in the 3’-end portion of the genome encoding for the structural proteins (capsid, movement and aphid-transmission proteins, respectively), while the 5’-end region of the genome encodes replication-related proteins (ORF1 and ORF2) and also a gene (ORF0) encoding the RNA silencing suppression protein P0. In general, the genome sequences of resistance breaking CLRDV isolates are very similar to CLRDV isolates from susceptible plants [3]. For example, the degree of sequence identity in all proteins encoded by ORFs 1 to 5 is greater than 93 % between two resistance breaking CLRDV isolates (Ima2 and Acr3) and two non-resistance breaking ones (PV1 and ARG) in cotton plants. However, when the identities among P0 proteins are compared, the diversity is consistently higher, with identity numbers ranging from 85.8 to 86.6 % among the four isolates [3].

The P0 protein from several members of the genera *Polerovirus* and *Enamovirus,* family *Luteoviridae*, are known to be involved in the suppression of plant’s anti-viral defense mechanisms at variable degrees, depending on the species and isolates [5–11]. P0’s silencing suppression activity is mediated by promoting the destabilization of ARGONAUTE (AGO) proteins, key players in RNA silencing mechanisms [8, 12–15]. In plants, the RNA silencing pathway is triggered by double stranded RNAs (dsRNAs), which are processed by Dicer-like enzymes into small RNAs ranging from 20 to 24 nucleotides [16]. Viral-derived small interfering RNAs (siRNAs) produced during infections are readily recruited by AGO-containing RNA-induced silencing complexes (RISC) and used by the machinery to degrade viral genomic and sub-genomic sequences, being therefore an efficient anti-viral defense mechanism [17]. AGO is an important component of the machinery, since it directly binds to siRNAs and guide RISC to target RNAs. Viral RNA degradation may take place either at locally infected cells or at distal tissues, by the systemic movement of silencing signals [18]. A plethora of evolutionary unrelated viral proteins has evolved to cope with the anti-viral RNA silencing process. The P19 proteins from tombusviruses are one of the best characterized suppressors. P19 proteins are able to bind to sRNAs, preventing their loading into RISC [19]. Similarly, by degrading AGO proteins, P0 proteins are able to suppress the plant’s anti-viral defense, allowing the infection to proceed. P0 proteins probably exert their activity through an F-box-dependent interaction with homologs of the S-phase kinase-related protein 1 (SKP1) ASK1 and ASK2 [20]. SKP1 is a core component of the SKP1/Cullin1/F-box (SCF) family of E3 ubiquitin ligases that mediate the ubiquitination of diverse regulatory and signaling proteins [21]. Point mutations in P0’s F-box motif may abolish its interaction with SKP1 and consequentially decreasing AGO destabilization and viral pathogenesis [8, 9, 11, 20]. However, P0’s activity is insensitive to proteasome inhibitors and the viral protein probably operates by hitchhiking cellular autophagy pathways endogenously used to modulate AGO homeostasis [13–15]. This model is supported by the increased accumulation of AGO proteins in the presence of autophagy inhibitors and by its co-localization with autophagic vesicles [14].

Recently, the P0 protein from an Argentinian isolate of CLRDV (P0^CL-ARG^) has been characterized as a RNA silencing suppression protein [10]. The level of both local and systemic silencing suppression observed in P0^CL-ARG^ seems to be low when compared to other members of the group. Almost no suppression of systemic silencing is observed for P0^CL-ARG^, in line with what has been previously found for the P0 proteins from other members of the family [5, 10, 22]. However, it’s known that P0 silencing suppression activity can vary even among closely related viruses. For example, European isolates of beet mild yellowing virus vary greatly in their ability to suppress local silencing [7]. Here, the local and systemic silencing suppression activities of P0 proteins from CLRDV isolates collected in different parts of Brazil, including CBD resistant and susceptible cotton varieties, were assessed and compared to P0^CL-ARG^. Results indicate that silencing suppression capabilities are strain-specific and that strength of local and systemic silencing suppression is not correlated in CLRDV P0 proteins.

## Results and discussion

### Sequence diversity among CLRDV P0 proteins

**Fig. 1:**
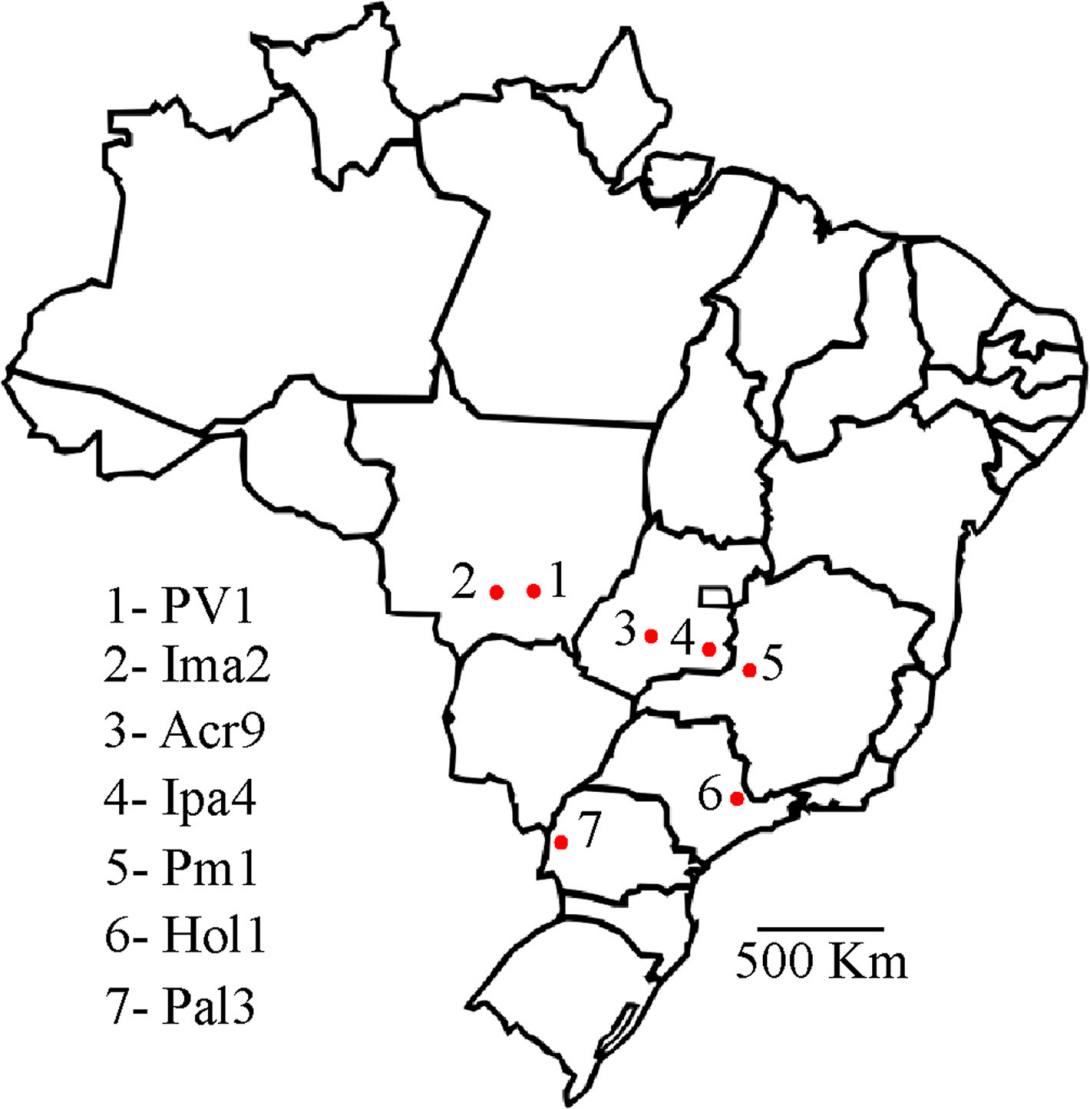
Map of Brazil showing sites where cotton plants were harvested for the study. The numbered red dots in the map indicate the different cotton leafroll dwarf virus isolates harvested

When the genomic sequences of four previously identified CLRDV isolates were compared, significant differences were only observed in the P0 coding sequence [3]. In order to better characterize the sequence diversity of P0 proteins among CLRDV isolates, cotton plants from different varieties, displaying typical or atypical CBD symptoms were collected from different parts of Brazil. Sampled areas covered the main cotton producing areas of the country, most of them with significant geographical distance from each other (Fig. 1). In total, seven Brazilian CLRDV isolates (PV1, Ima2, Acr9, Ipa4, Pm1, Hol1 and Pal3) were analyzed and compared to the Argentinian isolate (ARG) [4] and also to the P0 from an Australian isolate of potato leafroll virus (P0^PL-AU^) [8] (Table 1). The genomes from two of the Brazilian isolates analyzed (PV1 and Ima2) have been previously obtained [3, 23]. Three CLRDV isolates (Acr9, Ima2 and Ipa4) were obtained from known cotton CLRDV-resistant varieties and are therefore treated as resistance-breaking isolates. Two isolates (Ima2 and Pm1) were obtained from plants showing atypical symptoms (Table 1).

**Table 1:** Brazilian isolates of cotton leafroll dwarf virus used in the study

Isolate	Location^a^	*G. hirsutum* cultivar	CBD resistance phenotype	Symptoms observed	Year
PV1	Primavera do Leste – MT	FM966	Susceptible	Typical	2004
Acr9	Acreuna – GO	CD406	Resistant	Typical	2006
Hol1	Holambra – SP	Nd	Nd	Typical	2007
Ima2	Campo Verde - MT	IAC25 RMD	Resistant	Atypical	2009
Ipa4	Ipameri - GO	Delta Opal	Resistant	Typical	2006
Pal3	Palotina - PR	CD034928	Nd	Typical	2006
Pm1	Patos de Minas - MG	Epamig1	Nd	Atypical	2007

^a^MT, Mato Grosso State; GO – Goiás State; SP – São Paulo State; PR – Paraná State; MG – Minas Gerais State

The amino acid sequence identity among CLRDV P0s varied from 85.5 % (P0^CL-Ima2^/P0^CL-Hol1^) to 98.88 % (P0^CL-Pm1^/P0^CL-Pal3^ and P0^CL-ARG^/P0^CL-Pal3^) (Table 2). The P0 sequences from the isolates Hol1, Pal3 and Pm1 are very close to the P0s from PV1 and ARG, the two isolates initially associated with CBD [1, 4], with identities ranging from 97.76 % to 98.88 % (Table 2). The isolates Acr9, Ima2 and Ipa4 also have a high sequence identity among them, ranging from 95.53 % to 97.2 %. The identity among P0 sequences from isolates Acr9, Ima2 and Ipa4, however, is lower than 90 % when compared to PV1 or ARG. The current taxonomic criteria in the family *Luteoviridae* states that viruses having amino acid divergence higher than 10 % in any protein sequence should be considered as different species [24]. In this line, the isolates Acr9, Ima2 and Ipa4 should be regarded as a new species associated with CBD. Since P0 sequences are the most variable sequences among poleroviruses, it has been recently proposed that viruses having high diversity in this region, but with amino acid identities higher than 90 % in all other proteins should be regarded as genotypes of the same species and not as a different one [25, 26]. This kind of analysis can only be made for the Ima2 isolate, the only one of the three with the genome fully sequenced [3]. But since the P0 sequences of the three isolates are very similar (Table 2), Acr9 and Ipa4 are probably also new genotypes of CLRDV, as previously stated for Ima2 [3].

**Table 2:** Percentage of amino acid identity among the viral isolates used in the study

P0^CL-Pal3^	X								
P0^CL-Pm1^	98.88	X							
P0^CL-PV1^	98.51	98.14	X						
P0^CL-Hol1^	98.51	98.14	97.76	X					
P0^CL-ARG^	98.88	98.51	98.14	98.14	X				
P0^CL-Ima2^	86.24	85.87	86.61	85.5	86.24	X			
P0^CL-Ipa4^	88.1	87.73	87.73	87.36	87.36	96.28	X		
P0^CL-Acr9^	88.1	87.73	87.73	87.36	88.1	95.53	97.02	X	
P0^PL-AU^	18.21	18.21	17.84	18.21	18.58	17.84	18.58	18.58	X
	P0^CL-Pal3^	P0^CL-Pm1^	P0^CL-PV1^	P0^CL-Hol1^	P0^CL-ARG^	P0^CL-Ima2^	P0^CL-Ipa4^	P0^CL-Acr9^	P0^PL-AU^

**Fig. 2:**
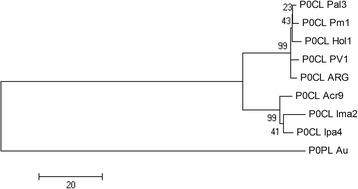
Neighbor joining phylogenetic tree of the cotton leafroll dwarf virus isolates used in the study. Tree was constructed based on P0’s amino acid sequence alignments. Numbers above the lines indicate the bootstrap scores with 1,000 replicates. Scale bar represents genetic distance. The Australian isolate of PLRV P0 was used as outgroup

The separation of the isolates in two groups is also supported by phylogenetic analysis. It has been shown that, when full genomic sequences are not available, P0-based phylogenies can correctly reconstruct the relatedness among poleroviruses [25]. A Neighbor-joining tree based on CLRDV P0s groups the isolates Hol1, Pal3 and Pm1 with the two CBD founding members PV1 and ARG, while the isolates Acr9, Ima2 and Ipa4 clearly branch out, forming a well-supported group (Fig. 2). Since the three divergent isolates were all obtained from formerly cotton resistant varieties (Table 1), the silencing suppression activities from all isolates were then scored and compared.

### Suppression of local silencing by CLRDV P0s

Previous data have shown that P0^CL-ARG^ is weak suppressor of local silencing when expressed in N*icotiana benthamiana* leaves [10]. The suppression assay is based on the *Agrobacterium*-mediated transient co-expression of mGFP5 and a candidate silencing suppression protein in mGFP5-expressing *N. benthamiana* leaves (transgenic line 16c) [27]. When no silencing suppression is observed, the transiently expressed GFP triggers a strong RNA silencing response that ultimately leads to RNA degradation from both stable and transient transgenes. However, in the presence of a silencing suppression protein, GFP degradation is prevented and both transgenes (stable and transient) are expressed, increasing total GFP levels. The silencing suppression assay can be easily monitored by hand UV lamps. In order to check whether the sequence diversity observed among the P0s could also reflect variable silencing suppression activities, the P0 from all seven Brazilian isolates and the Argentinian isolate were tested side-by-side in the 16c *N. benthamiana* line and compared to P0^PL-AU^ and P19, two known suppressors of local silencing. All genes were cloned into the pGWB417 binary vector [28], leading to a 35S-driven expression of Myc-tagged proteins.

**Fig. 3:**
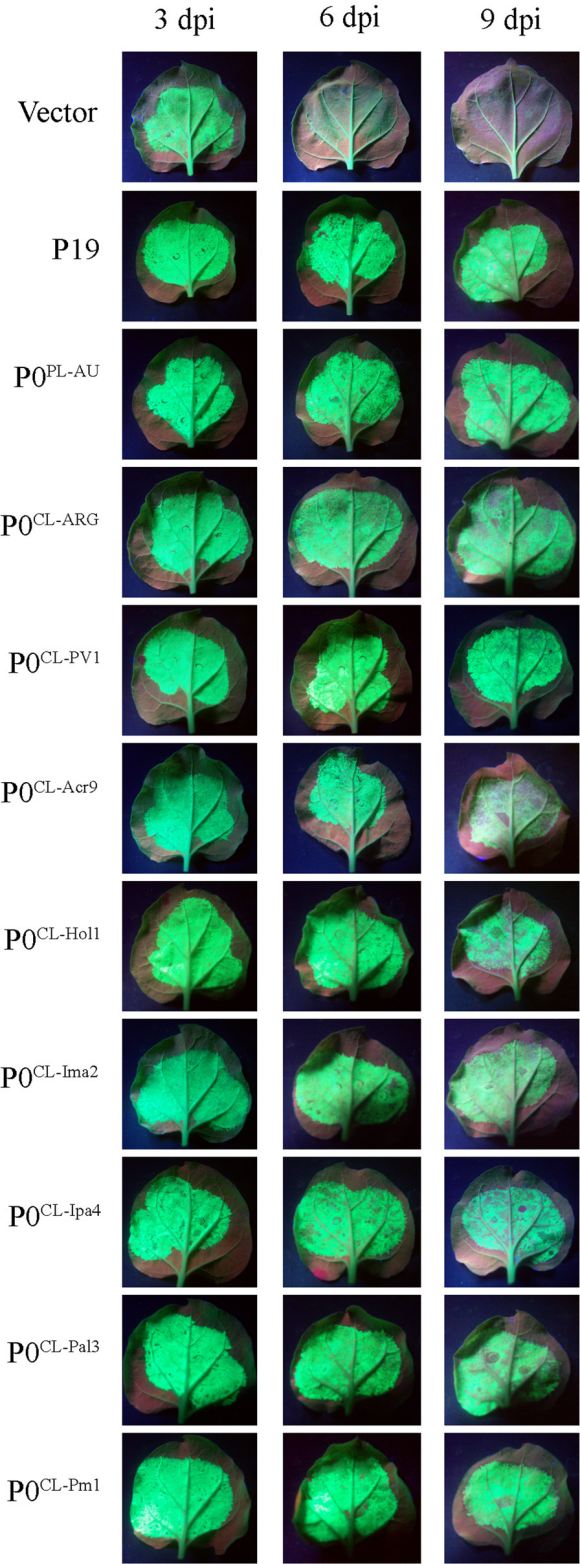
Suppression of local silencing by cotton leafroll dwarf virus P0s in *N. benthamiana* 16c plants. Transgenic *N. benthamiana* plants expressing GFP (line 16c) were co-infiltrated with *Agrobacterium* carrying plasmids to express GFP (silencing trigger) and candidate suppressor proteins (P0^CL-PV1^, P0^CL-Acr9^, P0^CL-Hol1^, P0^CL-Ima2^, P0^CL-Ipa4^, P0^CL-Pal3^ and P0^CL-Pm1^). GFP co-expressed with empty vector was used as negative control. P0^CL-ARG^, P0^PL-AU^ and P19 were used as positive controls in the assay. Pictures were taken at 3, 6 and 9 days post-infiltration (dpi)

**Fig. 4:**
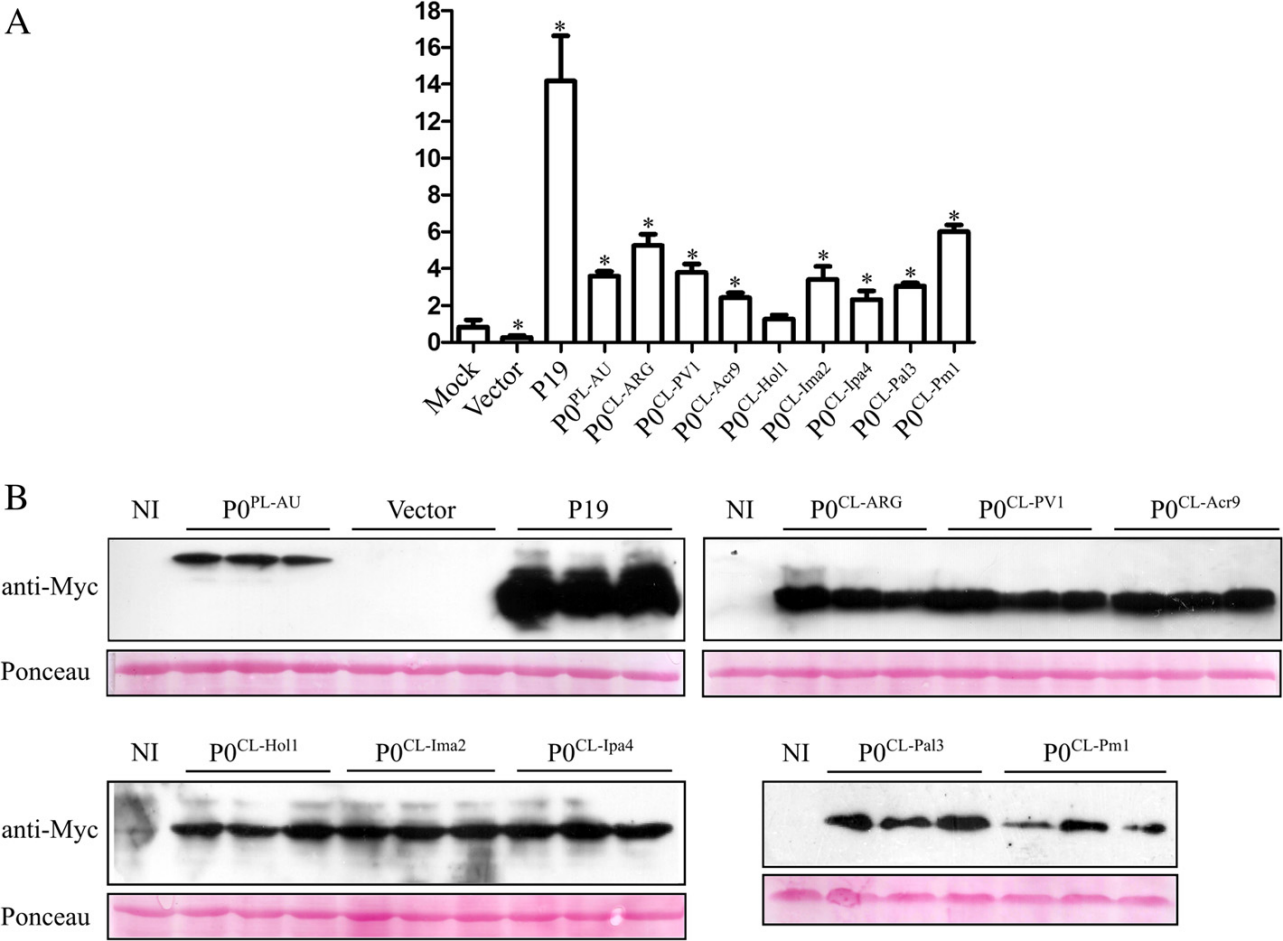
Accumulation of GFP mRNA and suppressor proteins in infiltrated *N. benthamiana* 16c leaves at 3 days after infiltration (dpi). **a** GFP levels in tissues co-infiltrated with cotton leafroll dwarf virus P0 proteins (P0^CL-ARG^, P0^CL-PV1^, P0^CL-Acr9^, P0^CL-Hol1^, P0^CL-Ima2^, P0^CL-Ipa4^, P0^CL-Pal3^ or P0^CL-Pm1^), potato leafroll virus P0 (P0^PL-AU^), empty vector or mock-inoculated were detected with real-time PCR. Error bars indicate standard deviation of GFP mRNA in three biological repeats. Normalized value obtained in the mock sample was arbitrary set to 1 and all the other values compared to it. Data was normalized with Ubi3 and EF-1 reference genes. Asterisks indicate values that are statistically different from the control mock construct, with p-values varying from 0.0002 to 0.0478. **b** Western blot showing the accumulation of suppressor proteins. Protein extracts from all the three biological replicates from each construct used for real-time PCR were run in SDS-PAGE, transferred to membranes and probed with Myc-tag specific antibodies. Gel loading was observed by Ponceau staining. Non-infiltrated plants (NI) were used as negative controls. Top left panel: accumulation of the control proteins P0^PL-AU^ and tombusvirus P19 and the negative control (infiltrated with the empty vector). Top right panel: accumulation of P0^CL-ARG^, P0^CL-PV1^ and P0^CL-Acr9^ proteins. Bottom left panel: accumulation of P0^CL-Hol1^, P0^CL-Ima2^ and P0^CL-Ipa4^ proteins. Bottom right: accumulation of P0^CL-Pal3^ and P0^CL-Pm1^ proteins

As expected, a red patch in the infiltrated area was observed when GFP was expressed in the absence of a silencing suppression protein (Fig. 3). Conversely, strong GFP fluorescence was observed in the presence of P19 or P0^PL-AU^ in all time-points analyzed (Fig. 3). All tested CLRDV P0s displayed obvious RNA silencing suppression activities. When scored based on GFP fluorescence, the levels of silencing suppression activity observed for the CLRDV P0s were similar to P0^PL-AU^, but significantly lower than the P19 suppressor protein even at 3 days post-infiltration (dpi) (Fig. 3). The observed GFP fluorescence at 3 dpi correlates well with the accumulation of GFP RNAs when checked by real-time PCR at this time-point in *N. benthamiana* 16c plants (Fig. 4a). GFP RNA levels in GFP/P19-infiltrated plants were approximately 14 times higher than in control mock-infiltrated 16c plants. However, the GFP RNA fold change in P0^PL-AU^ or CLRDV P0-infiltrated plants, varied from only 2 to 6 times the levels obtained in the same control condition (Fig. 4a). At later time-points, GFP fluorescence started to fade at infiltrated areas of P0^CL-Acr9^, P0^CL-Hol1^, P0^CL-Ima2^ and P0^CL-Ipa4^, indicating that those proteins are weaker suppressors than the other P0s (Fig. 3). The accumulation of the Myc-tagged suppressor proteins in the three biological replicates used for real-time PCR was checked by western blot using anti-Myc antibodies (Fig. 4b). Although all suppressors were expressed from the same vector background (pGWB417) [28], the P19 protein accumulated at levels consistently higher than the P0s. The strong suppression activity observed for P19 in the assay, therefore, might be correlated with its higher stability in *N. benthamiana* leaves. Since the P0s accumulated at similar levels, the observed differences in silencing suppression activities for those proteins might be due to functional divergence and not expression levels.

The fading phenotypes observed in 16c plants for the suppressor proteins P0^CL-Acr9^, P0^CL-Hol1^, P0^CL-Ima2^ and P0^CL-Ipa4^ were reproduced when the experiment was repeated in wild type plants (Additional file 1: Figure S1). When transiently expressed alone in wild type plants, the accumulation of GFP was lower than in the presence of the control strong suppressor P19, even at 3 dpi (Additional file 1: Figure S1). At 6 dpi, GFP was almost totally silenced when expressed alone, contrasting to what was observed in the presence of control constructs (P19 or PL^P0-AU^) or any of the CLRDV P0s. From 6 dpi onwards, as mentioned before, GFP levels started to fade in the presence of P0^CL-Acr9^, P0^CL-Hol1^, P0^CL-Ima2^ and P0^CL-Ipa4^, also indicating that those proteins are not able to suppress GFP silencing in *N. benthamiana* leaves for long periods. In all time-points analyzed, GFP accumulated at levels expressively higher when co-infiltrated with P19 than in the presence of any other P0, indicating that even the ones able to maintain GFP suppression at later times post-infiltration (P0^PL-AU^, P0^CL-ARG^, P0^CL-PV1^, P0^CL-Pal3^ and P0^CL-Pm1^) should be regarded as moderate suppressors compared to the control used in the assays.

It has been previously shown that P0s depend on the presence of a F-box-like motif to exert their silencing suppression activity [8, 9, 11, 20]. The hallmark amino acids LPxx(L/I)x^10–13^P could be found in all CLRDV P0s tested (Additional file 2: Figure S2). However, isoleucine is changed to an amino acid with similar biochemical properties (valine) in the three resistance-breaking CLRDV isolates (Acr9, Ima2 and Ipa4). The ring structure amino acids known to affect local silencing suppression activity of melon aphid-borne yellows virus P0 are also present and conserved among the CLRDV P0s (Additional file 2: Figure S2) [9]. Therefore, the local silencing suppression variability observed among CLRDV P0s is probably associated with alternative functional residues.

### Suppression of systemic silencing by CLRDV P0s

Viral siRNAs produced in infected cells may also move systemically through vascular tissues to reach other parts of the plants [18]. In the *N. benthamiana* 16c assay, systemic silencing can be visualized by the appearance of red-silenced areas especially around the veins of newly developed leaves. Eventually, silencing signals may spread throughout the leaves, producing completely silenced plants. The systemic silencing suppression activities of all CLRDV P0s were scored and compared to the strong systemic suppressor P0^PL-AU^ [8]. At 16 dpi, 15 out of 20 plants assayed showed systemic silencing when infiltrated with the GFP silencing-trigger construct in the absence of any suppressor protein and almost 100 % of the plants were silenced by 20 dpi (Table 3). As expected, P0^PL-AU^ completely blocked the spread of silencing signals as no silenced plants were observed at 16, 20 or 29 dpi. However, the suppression of systemic silencing mediated by the CLRDV P0s varied among the different isolates. In our experimental conditions, only 3 plants out of 20 were silenced at 16 dpi when co-infiltrated with P0^CL-ARG^ (Table 3). The number of silenced plants, however, increased to 7 and 10 out of 20 at 20 dpi and 29 dpi, respectively, indicating that P0^CL-ARG^ is a moderate suppressor of silencing signals (Table 3). This result contrast to what has been previously observed for P0^CL-ARG^, where 8 out 10 plants were already silenced by 15 dpi in the presence of the protein [10]. Since the spread of RNA silencing signals may be influenced by environmental conditions [29–31] and possibly by the number of infiltrated leaves, concentration and strain of *Agrobacterium* used in the assay and vector background, the difference in the systemic silencing activity observed for P0^CL-ARG^ might be due to different experimental settings.

**Table 3:** Proportion of plants showing systemic silencing at 16, 20 and 29 days post-infiltration (dpi)

Infiltration	16 dpi	20 dpi	29 dpi
GFP + Vector	15/20	19/20	19/20
GFP + P0^PL-AU^	0/20	0/20	0/20
GFP + P0^CL-PV1^	0/20	0/20	0/20
GFP + P0^CL-ARG^	3/20	7/20	10/20
GFP + P0^CL-Acr9^	5/20	7/20	7/20
GFP + P0^CL-Hol1^	0/20	1/20	2/20
GFP + P0^CL-Ima2^	7/20	9/20	11/20
GFP + P0^CL-Ipa4^	0/20	0/20	0/20
GFP + P0^CL-Pal3^	0/20	0/20	0/20
GFP + P0^CL-Pm1^	1/20	1/20	1/20

In line with what has been observed for P0^CL-ARG^, the P0 proteins P0^CL-Acr9^ and P0^CL-Ima2^ were also moderate suppressors of systemic silencing (Table 3). However, the P0 proteins from five isolates (PV1, Hol1, Ipa4, Pal3 and Pm1) efficiently blocked the spread of systemic silencing signals, with a silencing suppression activity similar to P0^PL-AU^ (Table 3). During the experiments, 70 % of the plants infiltrated with suppressor proteins P0^CL-Pal3^ and P0^CL-Pm1^ and 50 % of P0^PL-AU^-infiltrated ones displayed strong necrotic lesions at late times after infiltration, most of them starting at 10 dpi (data not shown). Therefore, the suppression of systemic silencing by those proteins could have also been influenced by the induced cell death. Similar necrotic phenotypes have also been observed for P0^PL-AU^ [8] and for the P0s from sugarcane yellow leaf virus and beet western yellows virus [6].

### AGO destabilization by CLRDV P0s

**Fig. 5:**
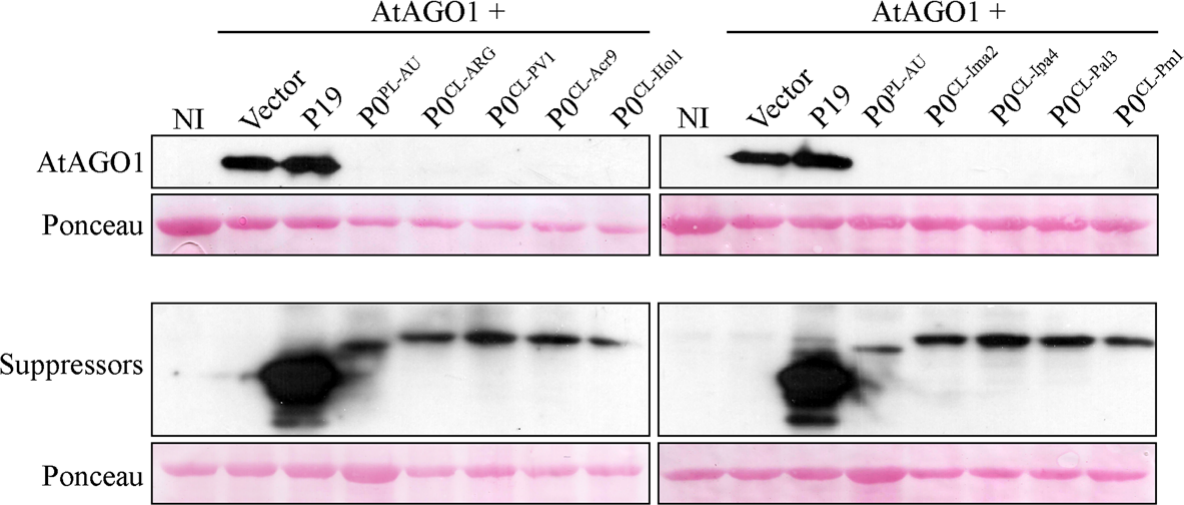
Accumulation of a Myc-tagged version of the *A. thaliana* AGO1 protein transiently expressed in *N. benthamiana* leaves in the presence or absence of P0 proteins. Myc-tagged versions of cotton leafroll dwarf virus P0s (P0^CL-ARG^, P0^CL-PV1^, P0^CL-Acr9^, P0^CL-Hol1^, P0^CL-Ima2^, P0^CL-Ipa4^, P0^CL-Pal3^ or P0^CL-Pm1^) and potato leafroll virus P0 (P0^PL-AU^) were used in the assay. Non-infiltrated plants (NI) were used as negative controls and AtAGO1 infiltrated with the empty vector or with a Myc-tagged P19 were used as positive controls. Membranes were probed with anti-Myc antibodies. Ponceau staining was used as a loading control. All co-infiltrations were performed in the presence of P19 (without tag) to stabilize mRNA *Agrobacterium*-mediated experiments

The P0 proteins from some members of the family *Luteoviridae* are able to destabilize the expression of AGO proteins [8, 11–15]. However, the activity of CLRDV P0 in AGO decay has never been tested. For that, a Myc-tagged version of the *Arabidopsis thaliana* AGO1 protein, known to be involved in several RNA silencing pathways, including anti-viral defense [32, 33], was transiently expressed via *Agrobacterium* infiltration in wild type *N. benthamiana* leaves in the presence or absence of different P0s or control constructs. The accumulation of AtAGO1-Myc protein was detected by anti-myc antibodies when co-expressed without P0 suppressors or in the presence of P19 (Fig. 5). All P0s, including P0^PL-AU^, the seven Brazilian isolates and the Argentinian isolate of CLRDV were able to strongly decrease the AtAGO1-Myc levels when co-expressed. Therefore, the local and systemic silencing differences observed among the isolates might be due to the regulation of still unknown cellular proteins by P0, possibly other AGO members [8], or due to small differences in AGO1 accumulation that are not detected due to western blot resolution limits.

## Conclusions

Our results indicated a high diversity among P0 proteins from Brazilian and Argentinian isolates of CLRDV, a virus associated with CBD. All CLRDV P0 proteins analyzed were able to mediate AtAGO1 decay, however, variable silencing suppression activities were observed, probably reflecting their sequence diversity. P0^CL-ARG^ was a moderate silencing suppressor of both local and systemic silencing in our experiments, when compared to the positive control constructs used in the assays (Figs. 3, 4 and Additional file 1: Figure S1). Three proteins (P0^CL-PV1^, P0^CL-Pal3^ and P0^CL-Pm1^) were also moderate suppressors of local silencing, but strong suppressors of systemic silencing. Four other proteins behaved as weak suppressors of local silencing. Contrasting to control constructs (P19 and P0^PL-AU^) and to other CLRDV P0s, those four proteins (P0^CL-Acr9^, P0^CL-Ima2^, P0^CL-Hol1^ and P0^CL-Ipa4^) could not support GFP suppression for long periods when assayed in the mGFP5-expressing *N. benthamiana* 16c line (Fig. 3) or in wild type plants (Additional file 1: Figure S1). GFP levels clearly started to fade in the presence of those proteins from 6 dpi onwards (Fig. 3 and Additional file 1: Figure S1). However, two of the weak local silencing suppressor proteins (P0^CL-Hol1^ and P0^CL-Ipa4^) were able to almost completely block the spread of systemic silencing signals when assayed in 16c transgenic lines. Despite of their weak local silencing, P0^CL-Hol1^ and P0^CL-Ipa4^ are as strong as the control P0^PL-AU^ protein in suppressing systemic silencing. It’s tempting to speculate therefore that the strength of local and systemic silencing suppression activity might be genetically unlinked in P0 proteins. Furthermore, these data indicate that the silencing suppression capabilities of the distinct CLRDV P0 proteins are not directly linked to their genetic diversity.

## Methods

### Plant material and DNA constructs

*Gossypium hirsutum* plants belonging to at least six cultivars (FM966, CD406, CD034928, IAC25 RMD, Delta Opal and Epamig1) were collected in five different States of Brazil (Goiás, Mato Grosso, Minas Gerais, Paraná and São Paulo) (Table 1 and Fig. 1). The Ima2 isolate was collected in Campo Verde – Mato Grosso, but passed through the IAC24 RMD cotton variety at Instituto Matogrossense do Algodão (Primavera do Leste – Mato Grosso) before being sent for analysis [34]. Harvesting and maintenance of plants were performed according to Brazilian rules (MP 2.186-16/2001). Total RNA of all plants were extracted using Qiagen Plant RNA kit and 2.5 μg were used to prepare cDNAs with the O5R2 primer (5’-GCAACCTTTTATAGTCTCTCCAAT-3’), which anneals in the middle of CLRDV ORF5. The ORF0 sequences from all Brazilian isolates were amplified with primers CLP0_F (5’-CACCATGTTGAATTTGATCATCTGCAG-3’) and CLP0_R (5’-ACTGCTTTCTCCTTCAC-3’) and cloned into pENTRY-D-TOPO (Invitrogen). The ORF0 of the Argentinian isolate of CLRDV [GenBank: NC_014545.1] was synthesized and cloned into a pUC plasmid by the Blue Heron Biotechnology Inc (USA). The Argentinian ORF0 [10] and the P19 coding sequence [35] were amplified with primers CLP0_TOPO_F/CLP0_R and P19_TP_F (5’-CACCATGGAACGAGCTATACAAGGAAACG -3’)/P19_R (5’-TTACTCGCTTTCTTTTTCGAAGG-3’), respectively, and also cloned into pENTRY-D-TOPO (Invitrogen). All amplifications were performed with the Phusion High Fidelity Polymerase (NEB). Entry vectors containing the *Arabidopsis thaliana* Ago1 coding sequence [TAIR: AT1G48410] and the P0 from the Australian isolate of PLRV [GenBank: D13953.1] were described previously [8].

Genes in entry gateway clones were sequenced in both directions in automated ABI sequencers through dye terminator cycle method, using primers annealing in vector sequences. The accession numbers for the new P0 sequences obtained here are: [GenBank:KR185733] (isolate Acr9), [GenBank:KR185734] (isolate Pm1), [GenBank:KR185735] (isolate Hol1), [GenBank:KR185736] (isolate Ipa4), [GenBank:KR185737] (isolate Pal3). All genes in entry vectors were transferred through LR reactions to the binary destination vector pGWB417 [28], resulting in 35S-driven, Myc-tagged proteins when expressed in plants.

### Sequence analysis

Multiple sequence alignments of deduced amino acid sequences were performed with ClustalW2 (http://www.ebi.ac.uk/Tools/msa/clustalw2/) and phylogenetic reconstructions were performed with the MEGA 4 software [36]. Trees were constructed by the neighbor-joining (NJ) method [37], with the pair-wise deletion option and number of differences matrix.

### Agroinfiltration

*Agrobacterium tumefaciens,* strain GV3101, were infiltrated in *Nicotiana benthamiana* leaves as described previously [38]. Cells were individually diluted to an optical density of 1.0 at 600 nm before mixing the cultures. Leaves were infiltrated in the abaxial surfaces with needleless syringes and the infiltrated plants were incubated in growth chambers with a 16-hour photoperiod at 24 °C.

For the local silencing suppression assay, three leaves of 5-weeks-old mGFP5-expressing *N. benthamiana* plants (wild type or 16c line) [27] were co-infiltrated with equal volumes of *A. tumefaciens* harboring plasmids expressing mGFP5 and pGWB417 with or without candidate silencing suppressor genes. For the systemic silencing suppression assay, only one leaf of 3-weeks-old *N. benthamiana* 16c plants were co-infiltrated with equal volumes of *A. tumefaciens* expressing mGFP5 and pGWB417 (negative control) or with pGWB417 expressing candidate silencing suppressor genes. GFP fluorescence was observed under a long-wavelength UV lamp and the number of plants having systemic silencing scored in different time-points.

For the AGO1 destabilization assay, three leaves of 5-week-old wild type *N. benthamiana* plants were infiltrated with *A. tumefaciens* harboring plasmids pJL3:P19 [35], pGWB417-AtAGO1-MYC, and pGWB417 with or without candidate silencing suppression genes in a proportion of 30 %, 35 % and 35 %, respectively. The infiltrated leaves were collected 4 days after infiltration.

### qRT-PCR

Total RNA from approximately 100 mg of infiltrated *N. benthamiana* leaf tissue was extracted using the Plant RNA Purification Reagent (Invitrogen) according to manufacturer’s instructions. The quality of the RNA was checked by electrophoresis on 1.0 % agarose gels in 0.5X TAE buffer, and the RNA was quantified with NanoDrop 2000 spectrophotometer (Thermo Scientific). One microgram of total RNA was treated with DNase I (Promega) according to manufacturer’s instructions and used for cDNA synthesis with oligo d(T) primer and the SuperScriptIII (Invitrogen) enzyme, according to manufacturer’s instructions. Quantitative PCR reactions were performed in a total volume of 20 μL, using 5 μL of a 20-fold diluted cDNA. The amplification reactions were performed using the SYBR® Select Master Mix (Applied Biosystems), according to manufacturer’s instructions. Primers used for GFP were qmGFP5_F3 (5’-AGTGGAGAGGGTGAAGGTGATGC-3’) and qmGFP5_R4 (5’- TCCCTCAGGCATGGCGCTCTT-3’). The genes Ubiquitin3 (Ubi3) and Elongation factor-1 α (EF-1) were used as reference genes, with primers previously described [39]. Three biological and technical replicates were used for all samples. Quantification of GFP expression levels was performed using the comparative CT method (ΔΔCT) through the Miner and qBase softwares [40–42]. The t-student test was performed to compare the samples.

### Western blotting

Infiltrated *Nicotiana benthamiana* leaves were ground in liquid nitrogen and mixed with sample buffer (100 mM Tris [pH 6.8], 20 % glycerol, 4 % SDS, and 0.2 % bromophenol blue) containing 10 % β-mercaptoethanol [43]. Samples were then boiled at 90 °C for 10 min, and centrifuged for 5 min at 13,000 × g before loading on a gel. Extracts were run in 8 % SDS-PAGE gels for the detection of AtAGO1-Myc and in 12 % SDS-PAGE gels for detecting P19-myc, P0^PL-AU^-myc and P0^CLs^-myc with anti-myc antibody (1:2,000; Sigma, clone 9E10), followed by an anti-mouse HRP secondary antibody (1:5,000; Bio-Rad). Antibody–protein interactions were visualized using an enhanced chemiluminescence detection kit (GE Healthcare) according to the manufacturer’s instructions.

## Additional files

Additional file 1: Figure S1. Suppression of local silencing by cotton leafroll dwarf virus P0s in *N. benthamiana* wild type plants. *N. benthamiana* plants were co-infiltrated with *Agrobacterium* carrying plasmids to express GFP and candidate suppressor proteins (P0^CL-PV1^, P0^CL-Acr9^, P0^CL-Hol1^, P0^CL-Ima2^, P0^CL-Ipa4^, P0^CL-Pal3^ and P0^CL-Pm1^). GFP co-expressed with empty vector was used as negative control. P0^CL-ARG^, P0^PL-AU^ and P19 were used as positive controls in the assay. Pictures were taken at 3, 6, 9 and 12 days post-infiltration (dpi). (PNG 4388 kb) Additional file 2: Figure S2. Amino acid sequence alignment of cotton leafroll dwarf virus P0s. Sequences were aligned with ClustalW2 and conserved amino acids were shaded in black with Mview. The red bar highlights the conserved F-box-like domain. Functional ring structure residues described in Han *et al.*, 2010 are marked with asterisks. (PNG 1391 kb)
