# Supplementary figures and images for: Function and diversity of P0 proteins among cotton leafroll dwarf virus isolates
Source: Virol J. 2015 Aug 12;12:123. doi: 10.1186/s12985-015-0356-7 (PMC4531488; doi:10.1186/s12985-015-0356-7)

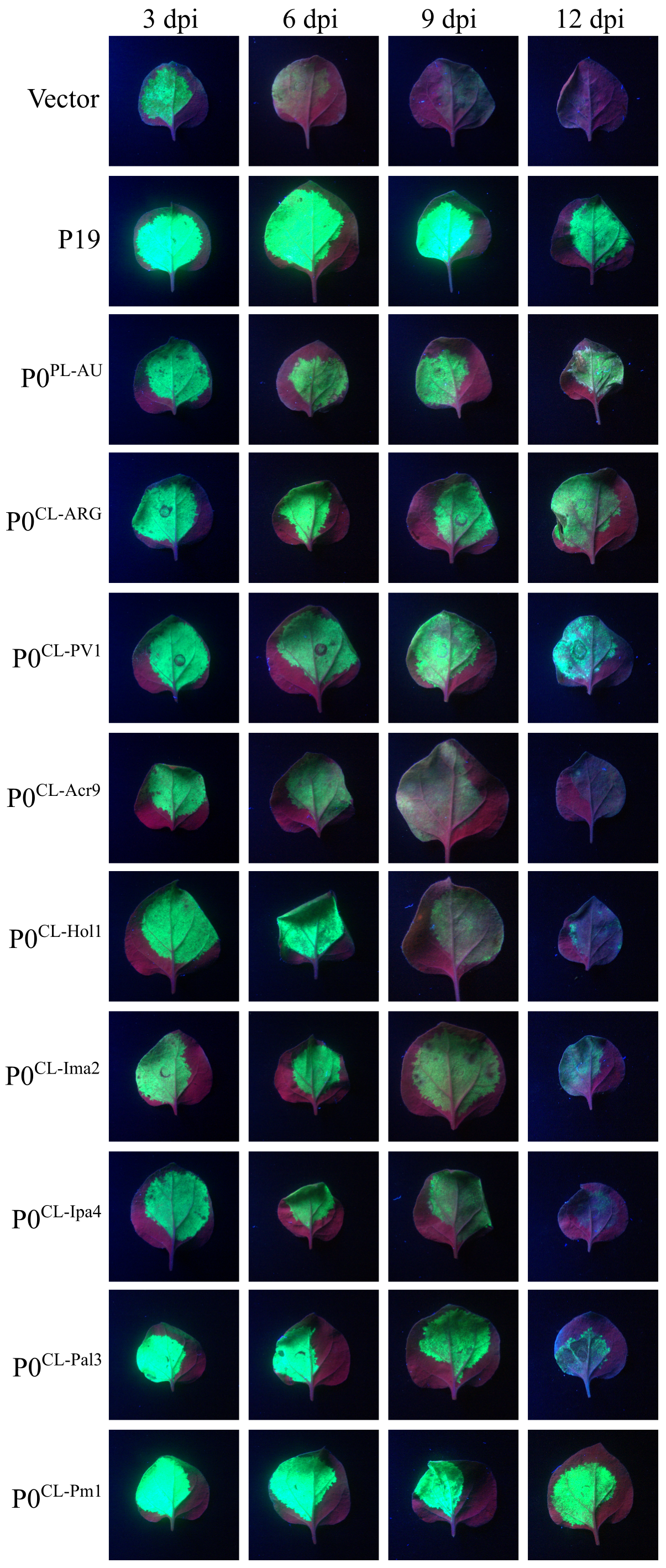

Supplement: Additional file 1: Figure S1. — Suppression of local silencing by cotton leafroll dwarf virus P0s in N. benthamiana wild type plants. N. benthamiana plants were co-infiltrated with Agrobacterium carrying plasmids to express GFP and candidate suppressor proteins (P0CL-PV1, P0CL-Acr9, P0CL-Hol1, P0CL-Ima2, P0CL-Ipa4, P0CL-Pal3 and P0CL-Pm1). GFP co-expressed with empty vector was used as negative control. P0CL-ARG, P0PL-AU and P19 were used as positive controls in the assay. Pictures were taken at 3, 6, 9 and 12 days post-infiltration (dpi). (PNG 4388 kb) [file 12985_2015_356_MOESM1_ESM.png]

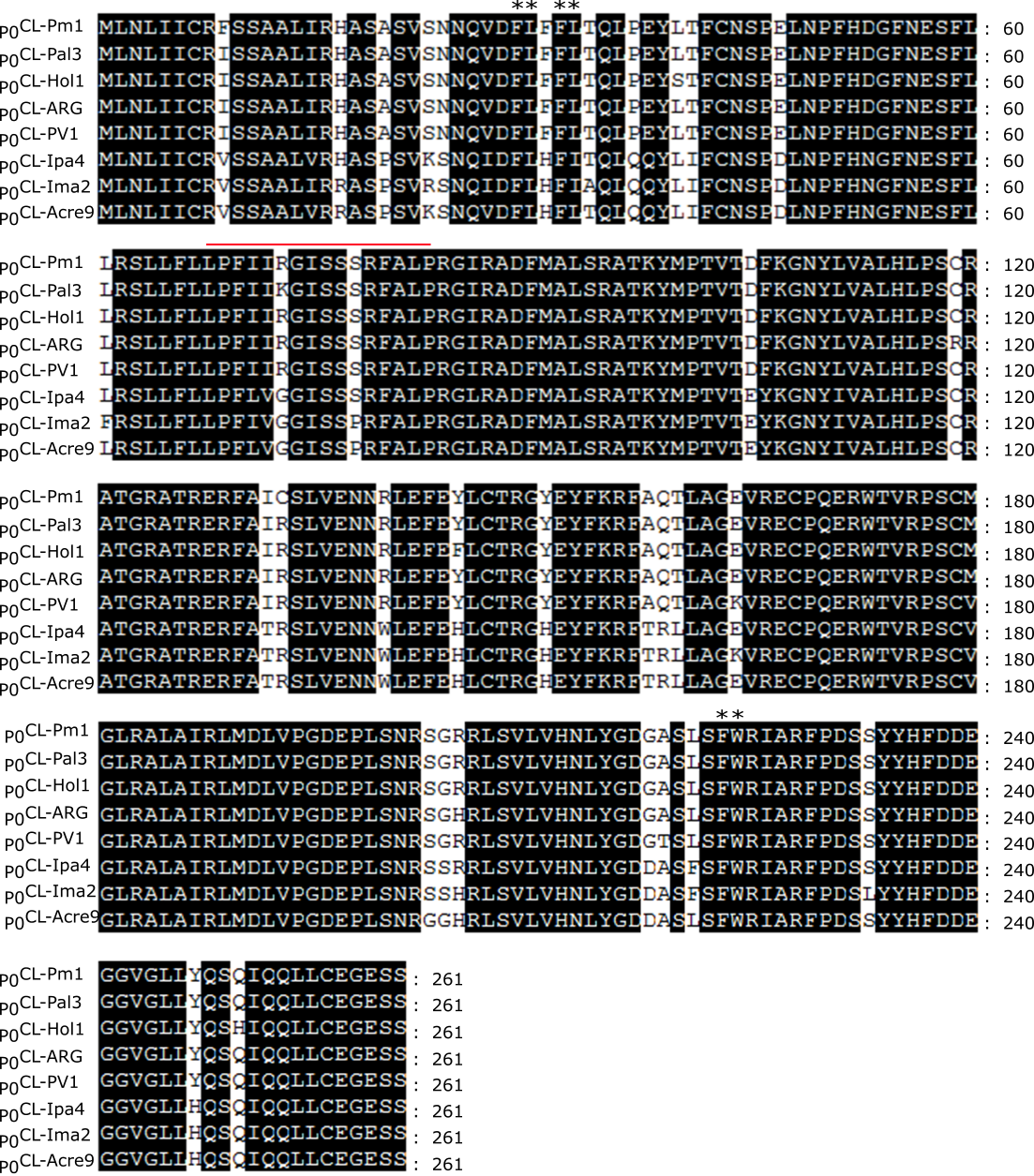

Supplement: Additional file 2: Figure S2. — Amino acid sequence alignment of cotton leafroll dwarf virus P0s. Sequences were aligned with ClustalW2 and conserved amino acids were shaded in black with Mview. The red bar highlights the conserved F-box-like domain. Functional ring structure residues described in Han et al., 2010 are marked with asterisks. (PNG 1391 kb) [file 12985_2015_356_MOESM2_ESM.png]
